# Female fertility and the zona pellucida

**DOI:** 10.7554/eLife.76106

**Published:** 2022-01-25

**Authors:** Paul M Wassarman, Eveline S Litscher

**Affiliations:** 1 Department Cell, Developmental, and Regenerative Biology Icahn School of Medicine at Mount Sinai One Gustave L. Levy Place New York United States; Icahn School of Medicine at Mount Sinai United States; Icahn School of Medicine at Mount Sinai United States

**Keywords:** zona pellucida, oogenesis, fertility, genetics, mutations

## Abstract

Fertility in female mammals, including mice and humans, is dependent on the presence of a zona pellucida (ZP) around growing oocytes and unfertilized eggs. A ZP is required to stabilize contacts between oocyte microvilli and follicle cell projections that traverse the ZP to form gap junctions that support the health of growing oocytes and developing follicles. In the absence of a ZP, due to inactivation or mutation of genes encoding ZP proteins, there is a loss of contacts between growing oocytes and neighboring follicle cells and a concomitant reduction in the production of ovulated eggs that results in female infertility.

## Introduction to the mammalian zona pellucida

**Figure 1.**
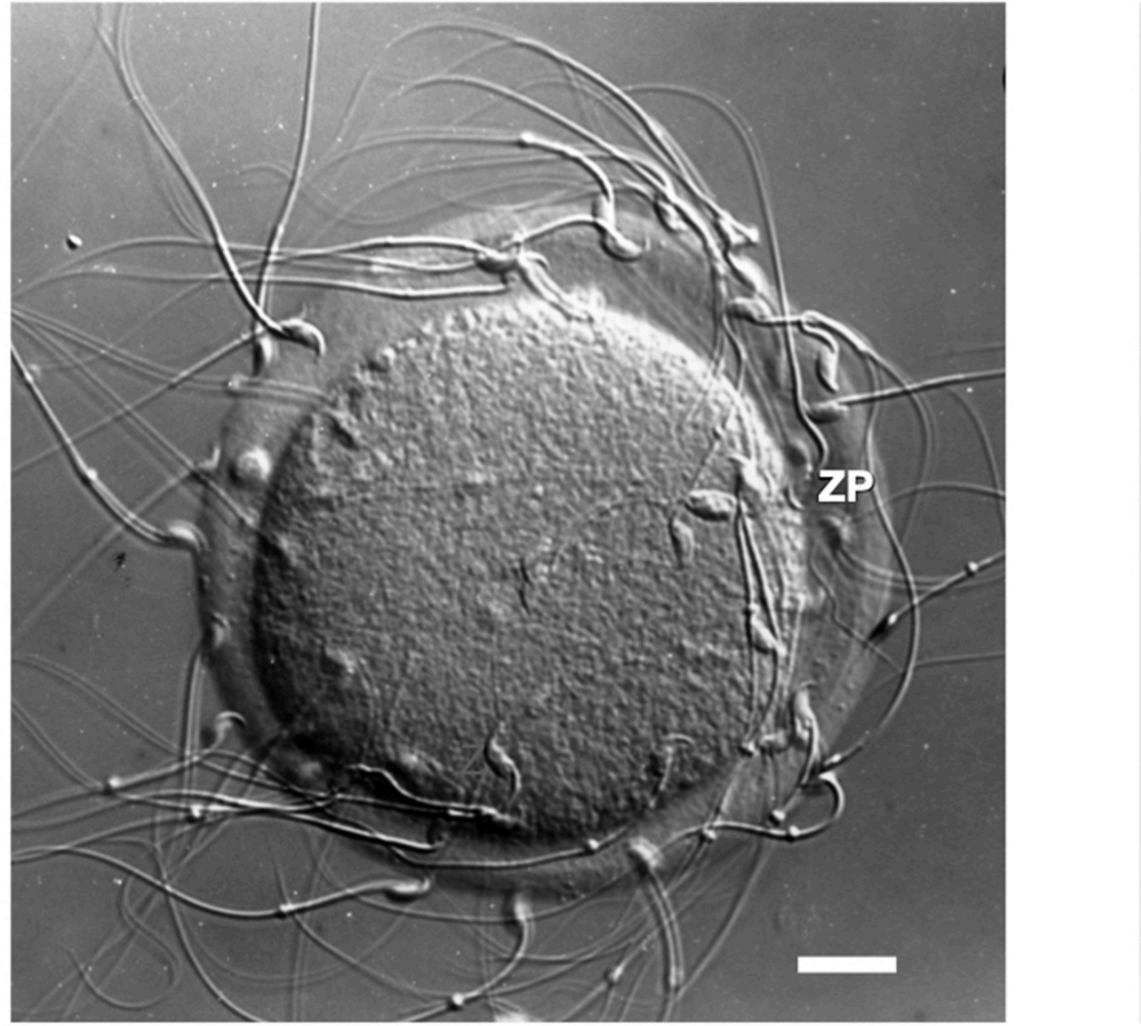
Photographic image of a light micrograph (Nomarski differential interference contrast) of an unfertilized mouse egg incubated in the presence of free-swimming sperm. Sperm are shown bound to the zona pellucida. Scale bar (≃1 cm) = ≃14 µm.

The zona pellucida (ZP) is a relatively thick extracellular matrix (ECM) that surrounds all mammalian oocytes, eggs, and preimplantation embryos, from monotremes that lay eggs to placental mammals (Wassarman and Litscher, 2016; Wassarman and Litscher, 2021). During oogenesis, small nongrowing oocytes that lack a ZP undergo tremendous growth to give rise to fully grown oocytes that have a ZP (Figure 1). Fully grown oocytes become unfertilized eggs when released or ovulated from the ovary into the fallopian tube where they may be fertilized by sperm. As oocytes begin to grow in the ovary the ZP first appears as diffuse fibrils in localized pockets around the surface of growing oocytes and shortly thereafter the fibrils coalesce to form a uniform ZP that continues to thicken as oocytes grow. Messenger RNA (mRNA) encoding ZP proteins is undetectable in nongrowing oocytes, but as soon as oocytes begin to grow ZP mRNA can be detected and increases to hundreds-of-thousands of copies in fully grown oocytes. A ZP consists of long interconnected fibrils laid down by a growing oocyte during oogenesis as ovarian follicles mature and prepare to ovulate an unfertilized egg.

During fertilization, free-swimming sperm recognize and bind to the ZP of unfertilized eggs (Figure 1) in a species-restricted manner, penetrate through the ZP, and fuse with egg plasma membrane to form a zygote. Shortly after an egg is fertilized, the ZP undergoes changes that affect its physical and biological properties that are important for preventing polyspermy and protecting preimplantation embryos in the female reproductive tract. For example, free-swimming sperm are unable to bind to the ZP of fertilized eggs or cleavage-stage embryos. The ZP of oocytes and eggs is a viscoelastic ECM whose viscosity increases several fold following fertilization (Kim and Kim, 2013). ECM elasticity, or stiffness, has been shown to dramatically affect a variety of cellular processes, such as adhesion of cells to ECM, cell spreading and migration, as well as cell growth, proliferation, and apoptosis (Chaudhuri et al., 2020). A ZP remains around cleavage-stage embryos until the expanded blastocyst stage (≃100–125 cells) when they consist of inner cell mass and trophectoderm and when they hatch from the ZP and implant in the uterus.

## Appearance of the ZP during mammalian oogenesis

**Figure 2.**
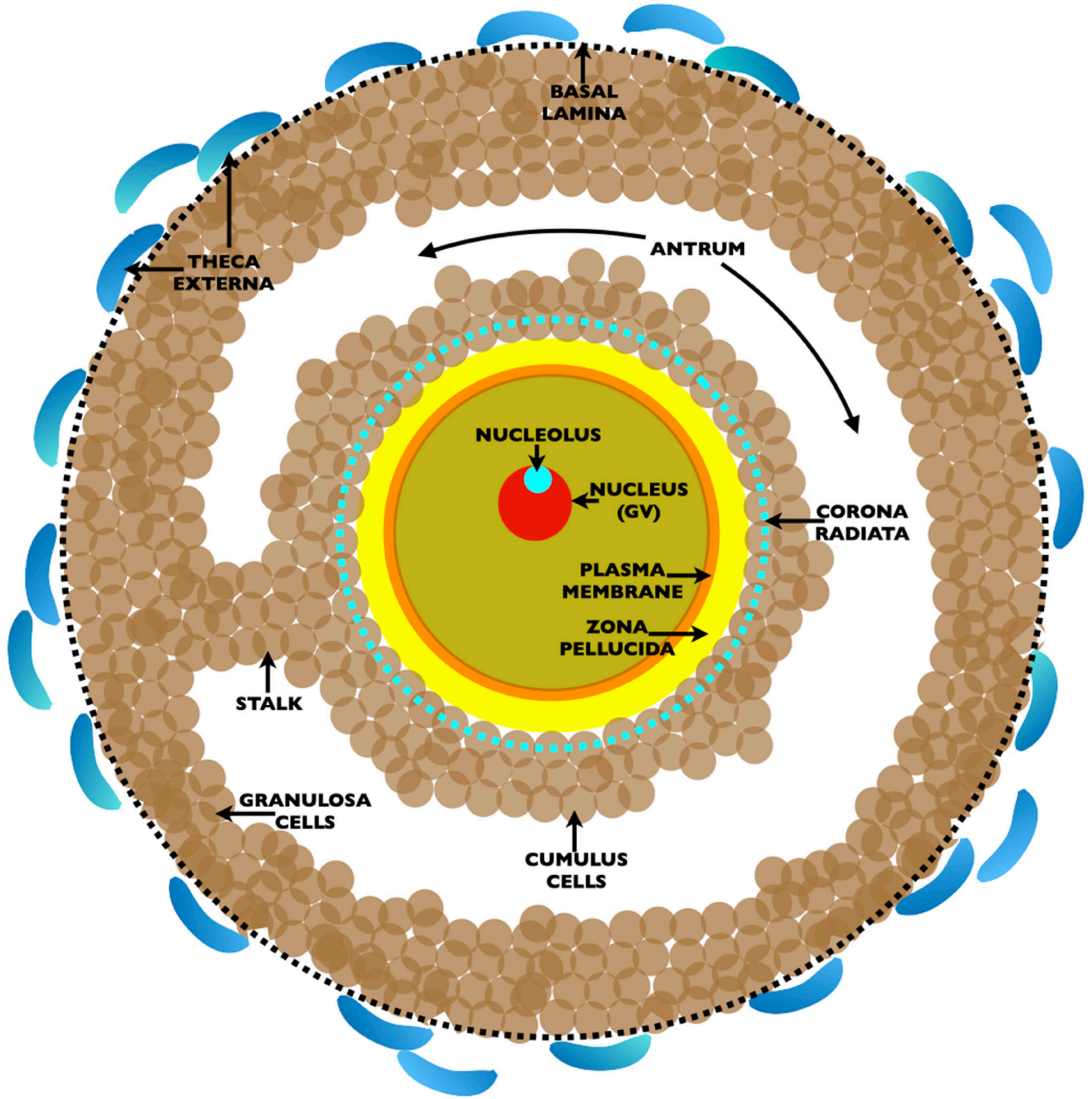
Drawing of an ovarian Graafian follicle prior to ovulation of an unfertilized egg. Shown is a fully grown oocyte (*pale green*) containing a nucleus/germinal vesicle (GV) (*red*), nucleolus (*turquoise*), plasma membrane (*orange*), and zona pellucida (*yellow*). Two-to-three layers of cumulus cells (*brown*) surround the oocyte and the innermost cumulus cells, the corona radiata, are indicated by a *dashed turquoise circle*. The oocyte is connected to the granulosa cells (*brown*) by a stalk and is located in a fluid filled cavity, the antrum. At the outermost region of the Graafian follicle is a basal lamina (*dashed black circle*) and outside of this are the theca externa cells (*blue*). At ovulation, oocytes arrested in metaphase II of meiosis are expelled from the Graafian follicle surrounded by two-to-three layers of cumulus cells. Oocytes resume meiosis, complete the first meiotic reductive division, called meiotic maturation, with separation of homologous chromosomes and emission of the first polar body, and become unfertilized eggs. At fertilization, eggs complete meiosis with separation of chromatids and emission of a second polar body. The sperm’s genome restores the fertilized egg to a diploid state.

Commencement of oocyte growth is regulated within the ovary, with the number of growing oocytes dependent on the size of the pool of nongrowing oocytes. Each nongrowing oocyte is surrounded by a few flat mitotic cells that differentiate and multiply profusely during and after oocyte growth and give rise to a very large Graafian follicle from which an unfertilized egg, surrounded by two-to-three layers of cumulus cells, is ovulated in response to hormones (Figure 2). Mouse and human Graafian follicles are ≃600 µm (≃50 thousand cells) and ≃20 mm (≃50 million cells) in diameter, respectively. The cohort of follicle cells, granulosa and thecal cells, that remain behind in the ovary following ovulation become an endocrine gland, the corpus luteum, that supports pregnancy by secreting progesterone.

**Figure 3.**
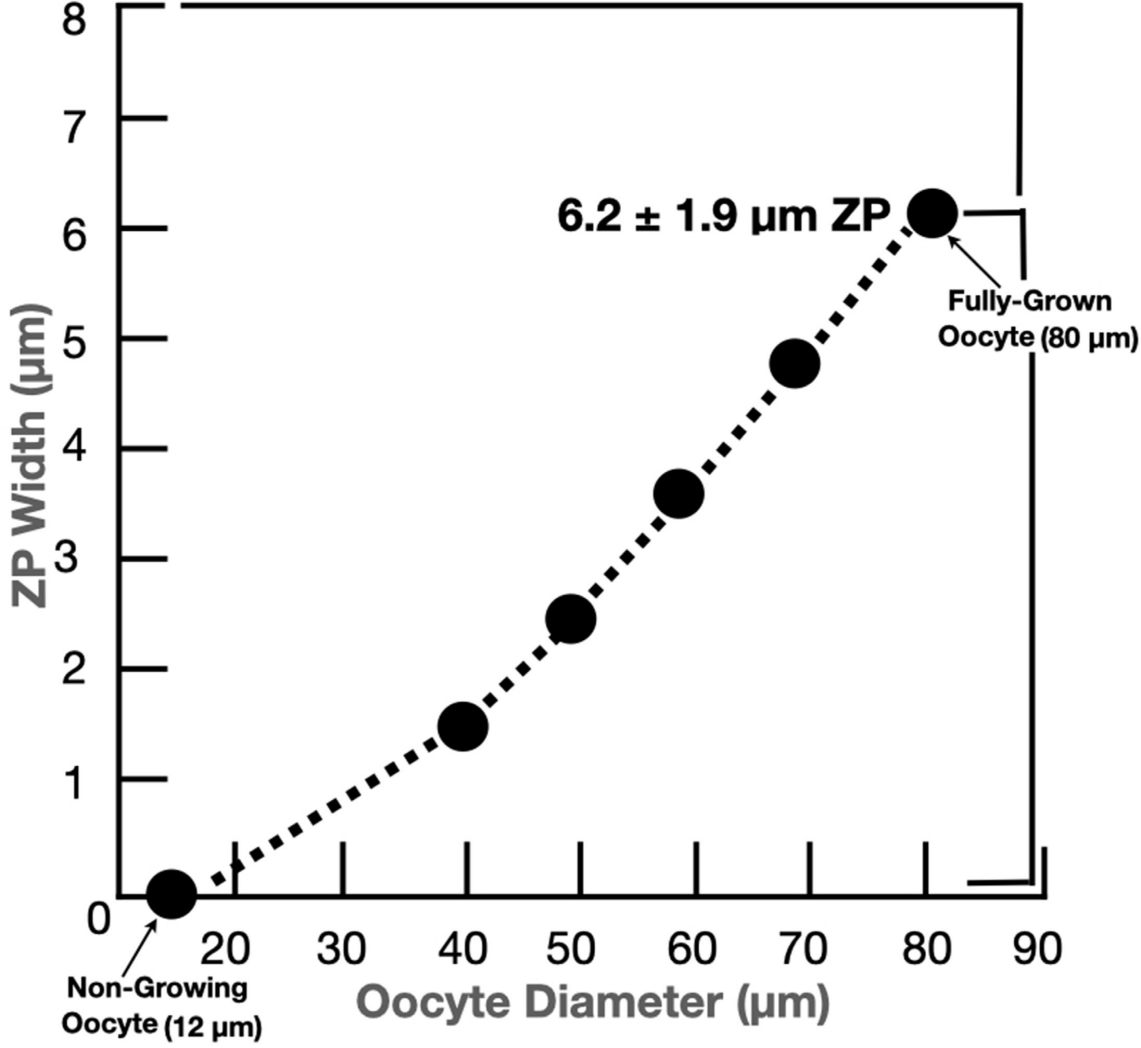
Graph depicting changes in the width of the mouse oocyte’s zona pellucida during growth of the oocyte. Mouse oocytes grow from ≃12 µm (nongrowing oocytes) to ≃80 µm in diameter (fully grown oocyte) over 2–3 weeks; this corresponds to more than a 300-fold increase in oocyte volume, from ≃0.9 to ≃300 pl, during oocyte growth. Concomitant with oocyte growth, the zona pellucida increases in width from zero for nongrowing oocytes to ≃6.2 ± 1.9 µm for fully grown oocytes.

Oogenesis begins in the outer layers of ovaries of mouse (≃day 13) and human (≃week 7) fetuses with formation of primordial germ cells that are converted into mitotic oogonia and then into oocytes at various stages of meiotic prophase (Austin, 1972; Conti and Chang, 2016; Larose et al., 2019). Oocytes progress through first meiotic prophase with pairing of homologous chromosomes, crossing-over, and recombination. At birth the ovary is populated with small, nongrowing oocytes that are arrested at the diplotene or dictyate stage of meiosis and lack a ZP. During each reproductive cycle in sexually mature females, oocytes grow, undergo many ultrastructural changes, acquire a thickening ZP (Figure 3), and are the sole source of unfertilized eggs. Mammalian eggs are ≃100 µm in diameter with not much more than a two-to-one variation in size for eggs from different mammals, with the exception of monotremes whose eggs are ≃25 times larger. The width of the ZP for eggs from different mammals varies from ≃2 to ≃22 µm (e.g., mouse ZP, ≃6 µm; human ZP, ≃18 µm).

## Oocyte–follicle cell communication in the ovary

The ovarian follicle is a functional syncytium that provides routes of bidirectional communication between oocytes and follicle cells, with follicle cells regulating oocyte growth and oocytes regulating follicle development (Sugiura and Eppig, 2005; Kidder and Vanderhyden, 2010). Narrow channels, called gap junctions, are present between growing oocytes and neighboring cumulus cells (corona radiata), as well as between all cumulus and granulosa cells (Goodenough and Paul, 2009). Each gap junction channel consists of two protein hexamers, called connexons, that consist of homomeric or heteromeric arrays of connexins, membrane proteins that bridge space between cells (Wei et al., 2004; Hervé, 2018). There are 20 or more different connexins in mice and humans. Gap junctions permit passage of electrical impulses and small molecules less than about 1000 molecular weight (e.g., amino acids, nucleotides, metabolites, ions, and second messengers) between neighboring cells and is regulated by several different proteins that associate with connexins (Alexander and Goldberg, 2003; Giepmans, 2004). Female mice that are homozygous nulls for either connexin-37 (gap junctions between oocytes and granulosa cells) (Simon et al., 1997) or connexin-43 (gap junctions between granulosa cells) (Ackert et al., 2001) fail to ovulate, are deficient in growing oocytes and multilayered follicles, and are completely infertile. This suggests that loss of gap junctions causes disruption of bidirectional communication between oocytes and follicle cells and results in infertility. Related to this is the recent report that interference with formation of oocyte microvilli by deletion of radixin, the microvilli-forming gene, leads to retardation of both oocyte growth and follicle development and to reduced fertility in mice (Zhang et al., 2021).

## Mouse and human ZP proteins

The mouse ZP is constructed of three glycosylated proteins, called mZP1–3, that are encoded by single-copy genes located on different chromosomes (Liang and Dean, 1992; Wassarman and Litscher, 2018). mZP2 and mZP3 are monomers and mZP1 is a dimer of identical polypeptides connected by an intermolecular disulfide. The human ZP is constructed of four glycosylated proteins, called hZP1–4, that are also encoded by single-copy genes located on different chromosomes (Gupta, 2018). hZP2 and hZP3 are monomers, hZP1 is a dimer of identical polypeptides connected by an intermolecular disulfide, and hZP4 is either a monomer or dimer held together by noncovalent interactions. Mouse and human ZP protein sequences are ≃63% identical and ≃84% similar and this suggests that the proteins perform identical functions (Chothia et al., 2003). In this context, it has been found that human ZP proteins can substitute for mouse ZP proteins in the mouse ZP (Rankin et al., 2003). In mouse oocytes mZP2 and mZP3 form heterodimers in the extracellular space which then polymerize into long fibrils that exhibit a structural repeat (≃15 nm) (Greve and Wassarman, 1985; Wassarman and Mortillo, 1991). The fibrils are crosslinked by mZP1 and possibly by many noncovalent interactions between fibrils. It can be assumed that ZP fibrils surrounding human oocytes are constructed in a similar manner during oogenesis.

**Figure 4.**
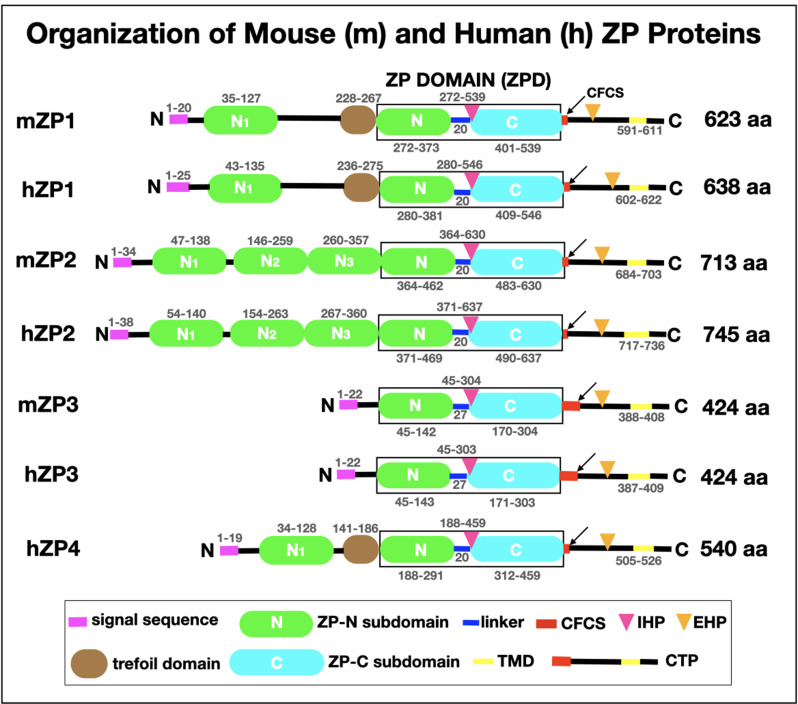
Schematic representation of the organization of mouse zona pellucida proteins, mZP1–3 (623, 713, and 424 amino acids, respectively), and human zona pellucida proteins, hZP1–4 (638, 745, 424, and 540 amino acids, respectively). In each case, the polypeptide contains a signal sequence (SS) at the N-terminus (*pink*), a ZP domain (ZPD; *black box*) consisting of ZP-N (*green*) and ZP-C (*turquoise*) subdomains and a short linker region (*blue*), and a consensus furin cleavage site (CFCS; *arrow*), transmembrane domain (TMD; *yellow*), and C-terminal propeptide (CTP). mZP1, hZP1, and hZP4 also have a trefoil domain (*brown*) adjacent to the ZPD. mZP1, mZP2, hZP1, hZP2, and hZP4 have one or three extra copies of the ZP-N subdomain (*green*) between the N-terminus of the polypeptides and the ZPD. The positions of the internal (IHP) and external (EHP) hydrophobic patches are indicated by *red* and *orange triangles*, respectively. The amino acid numbers for each region of the mouse and human zona pellucida polypeptides are indicated above and below the drawings of the polypeptides.

ZP proteins are synthesized as polypeptide precursors by growing oocytes and are processed by proteases prior to secretion into the extracellular space. Nascent mouse and human ZP polypeptides have many features in common (Figure 4). Among these features are: (1) A short signal sequence at the amino- terminus that targets nascent ZP proteins to the secretory pathway and is removed prior to secretion; (2) A ZP domain (ZPD) that consists of two subdomains, ZP-N and ZP-C, separated by a short linker region. The subdomains adopt immunoglobulin-like folds, a three-dimensional structure thought to have arisen ≃750 million years ago in sponges for use in vertebrate extracellular recognition systems (Barclay, 2003). The ZPD is essential for polymerization of extracellular proteins into fibrils and has been found in hundreds of proteins in a wide variety of organisms, from jellyfish to humans (Jovine et al., 2002; Jovine et al., 2005; Plaza et al., 2010; Litscher and Wassarman, 2015); (3) Internal and external hydrophobic patches (IHP and EHP, respectively) that interact with each other in ZP polypeptide precursors and prevent formation of fibrils in oocytes prior to proteolytic processing and secretion; (4) A carboxy-terminal propeptide (CTP) that contains a hydrophobic transmembrane domain used to insert nascent ZP proteins into secretory granule and plasma membrane and a short hydrophilic cytoplasmic tail that is removed by proteolytic cleavage at the consensus furin cleavage site (CFCS) during secretion; (5) mZP1, hZP1, and hZP4 also contain a trefoil domain (TD), possibly involved in crosslinking of ZP fibrils (Järvå et al., 2020), and one extra copy of subdomain ZP-N (N1) near their amino-terminus; mZP2 and hZP2 have three extra copies of ZP-N (N1–3) near their amino-terminus (Callebaut et al., 2007). Processing, secretion, and polymerization of nascent ZP proteins are regulated by polypeptide sequence elements such as the ZP-N, ZP-C, CFCS, CTP, IHP, and EHP (Zhao et al., 2003; Jovine et al., 2004; Jimenez-Movilla and Dean, 2011; Bokhove and Jovine, 2018; Wassarman and Litscher, 2018).

## Inactivation of mouse ZP genes causes infertility

To examine possible effects of the loss of ZP proteins on fertility of mice, mouse lines were established in which genes encoding mZP2 and mZP3 were inactivated by either homologous recombination or insertional mutagenesis (Table 1). Compared to wild-type females, homozygous nulls for genes encoding either mZP2 (mZP2^−/−^) or mZP3 (mZP3^−/−^) were completely infertile and exhibited smaller ovaries, reduced numbers of Graafian follicles and ovulated eggs, and fewer cumulus cells that were disorderly arrayed around oocytes (Liu et al., 1996; Rankin et al., 1996; Rankin et al., 2001; Wassarman and Litscher, 2018). These findings suggest that both mZP2 and mZP3 must be present to construct ZP fibrils and that the presence of a ZP around oocytes is essential for fertility in female mice. In this context, homozygous nulls for the gene encoding mZP1 (mZP1^−/−^) had an abnormal ZP that was insufficiently crosslinked such that cleavage-stage embryos were extremely fragile and often lost as they traversed the female reproductive tract on their way to the uterus (Rankin et al., 1999). Heterozygous nulls for the gene encoding mZP3 (mZP3^+/−^) were as fertile as wild-type females, but their eggs had a thin ZP (ave. width 2.7 ± 1.2 µm) and contained about one-half the amount of mZP2 and mZP3 found in ZP of eggs from wild-type mice (Wassarman et al., 1997). This suggests that ZP width is not a critical parameter for either binding of sperm to the ZP or fertilization of eggs.

**Table 1.** Phenotypes of ZP1, 2, 3 null female mice.

Genotype	Fertility	Zona Pellucida	References
*ZP1–3 wild-type*	Fertile	Normal	
*ZP1 homozygous-null*	Reduced	Abnormal	Rankin et al., 1999
*ZP2 homozygous-null*	Infertile	None	Rankin et al., 2001
*ZP3 homozygous-null*	Infertile	None	Rankin et al., 1996; Liu et al., 1996
*ZP3 heterozygous-null*	Fertile	Thin	Wassarman et al., 1997

## Mutation of human ZP genes causes infertility

It has been estimated that ≃50% of human infertility cases have a genetic component and that ≃50% are due to a female factor (Deshpande and Gupta, 2019). In 2005, a correlation was found between sequence variations in genes encoding human ZP proteins and fertilization failure (Männikkö et al., 2005). Subsequently, a mutated form of hZP1 was implicated in familial infertility in human females (Huang et al., 2014).

**Figure 5.**
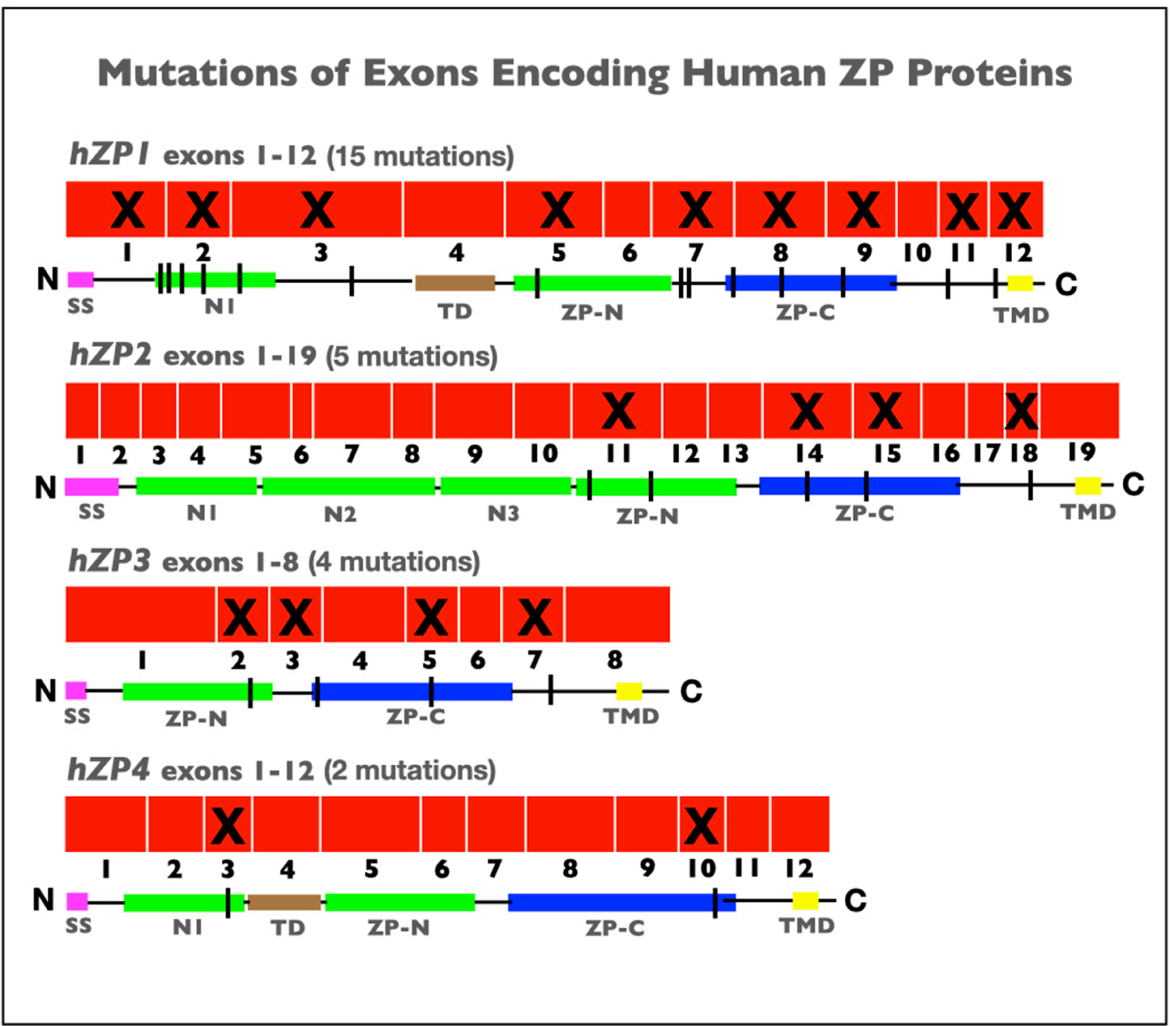
Schematic representations of mutations in exons encoding human zona pellucida proteins, hZP1–4. Shown is the organization of exons (*red*) for hZP1 (12 exons), hZP2 (19 exons), hZP3 (8 exons), and hZP4 (12 exons). Exons subject to mutations that caused infertility are marked by an X. Also shown are schematic representations of hZP1–4 polypeptides with the signal sequence (SS; *red*), trefoil domain (TD; *brown*), ZP domain (ZPD) consisting of ZP-N (*green*) and ZP-C (*blue*) subdomains, transmembrane domain (TMD; *yellow*), and extra copies of ZP-N subdomain (*green*) between the N-terminus of the polypeptides and the ZPD. The sites of mutations in the polypeptides are indicated by *black vertical lines*.

In recent years, more than two dozen cases of infertility in human female patients have been linked to point, missense, or frameshift mutations in human ZP genes encoding hZP1–4 (summarized in Wassarman and Litscher, 2021). Mutations were identified in nine exons encoding hZP1, four exons encoding hZP2, four exons encoding hZP3, and two exons encoding hZP4 (Figure 5). For several of the mutated genes, insertion of a premature stop-codon led to synthesis of truncated forms of human ZP proteins and to the absence of a ZP. More than a dozen of the mutations occurred in exons encoding the ZPD of hZP1–4, in either the ZP-N or ZP-C subdomains or in the linker region, and prevented construction of a ZP around oocytes. Similarly, for hZP1 and hZP4 several mutations occurred in exons encoding the N1 subdomain before the TD and resulted in either the presence of an abnormal ZP or no ZP around oocytes. As expected, the absence of ZP polypeptide sequence elements required for proteolytic processing, secretion, and assembly of nascent ZP proteins during oogenesis resulted in infertility. In every case, infertile patients had either abnormal eggs lacking a ZP or no eggs at all. As with female mice (Table 1), these findings with infertile human patients (Figure 5) suggest that the presence of a ZP around oocytes is essential for fertility in human females.

### Conclusions

The findings described here demonstrate that interference with construction of a ZP around growing oocytes, by either inactivation or mutation of genes encoding ZP proteins, results in female infertility in mice and humans. Failure to construct a ZP apparently interferes with bidirectional communication between oocytes and follicle cells in the ovary. Since the ZP is a viscoelastic ECM, it likely provides stability for contacts between projections from innermost cumulus cells (corona radiata) and oocyte microvilli that pass through the ZP and form the gap junctions shown to be essential for oocyte growth and follicle development (Gilula et al., 1978; Li and Albertini, 2013; Clarke, 2018; Baena and Terasaki, 2019). In the absence of a thickening ZP, interactions between growing oocytes and innermost cumulus cells may be unstable, leading to a significant reduction in the number of fully grown oocytes, ovulated eggs, and Graafian follicles and to female infertility. It is likely that further research with infertile women in in vitro fertilization (IVF) clinics will lead to identification of many additional mutations in genes encoding human ZP proteins.
